# Hippocampal Stratum Oriens Somatostatin-Positive Cells Undergo CB1-Dependent Long-Term Potentiation and Express Endocannabinoid Biosynthetic Enzymes

**DOI:** 10.3390/molecules24071306

**Published:** 2019-04-03

**Authors:** Lindsey N. Friend, Ryan C. Williamson, Collin B. Merrill, Scott T. Newton, Michael T. Christensen, Jake Petersen, Bridget Wu, Isaac Ostlund, Jeffrey G. Edwards

**Affiliations:** 1 Neuroscience Center, Brigham Young University, Provo, UT 84602, USA; lindseynfriend@gmail.com (L.N.F.); rwilliamson88@gmail.com (R.C.W.); scottnewton12@gmail.com (S.T.N.); 2 Department of Physiology and Developmental Biology, Brigham Young University, Provo, UT 84602, USA; collmerr@gmail.com (C.B.M.); michaeltchristensen@gmail.com (M.T.C.); peteyjr13@gmail.com (J.P.); jie_jiegr@hotmail.com (B.W.); isaac.ostlund@gmail.com (I.O.)

**Keywords:** anandamide, LTP, eCB, mGluR5, mGluR1, hippocampus, DAGLα, 12-lipoxygenase

## Abstract

The hippocampus is thought to encode information by altering synaptic strength via synaptic plasticity. Some forms of synaptic plasticity are induced by lipid-based endocannabinoid signaling molecules that act on cannabinoid receptors (CB1). Endocannabinoids modulate synaptic plasticity of hippocampal pyramidal cells and stratum radiatum interneurons; however, the role of endocannabinoids in mediating synaptic plasticity of stratum oriens interneurons is unclear. These feedback inhibitory interneurons exhibit presynaptic long-term potentiation (LTP), but the exact mechanism is not entirely understood. We examined whether oriens interneurons produce endocannabinoids, and whether endocannabinoids are involved in presynaptic LTP. Using patch-clamp electrodes to extract single cells, we analyzed the expression of endocannabinoid biosynthetic enzyme mRNA by reverse transcription and then real-time PCR (RT-PCR). The cellular expression of calcium-binding proteins and neuropeptides were used to identify interneuron subtype. RT-PCR results demonstrate that stratum oriens interneurons express mRNA for both endocannabinoid biosynthetic enzymes and the type I metabotropic glutamate receptors (mGluRs), necessary for endocannabinoid production. Immunohistochemical staining further confirmed the presence of diacylglycerol lipase alpha, an endocannabinoid-synthesizing enzyme, in oriens interneurons. To test the role of endocannabinoids in synaptic plasticity, we performed whole-cell experiments using high-frequency stimulation to induce long-term potentiation in somatostatin-positive cells. This plasticity was blocked by AM-251, demonstrating CB1-dependence. In addition, in the presence of a fatty acid amide hydrolase inhibitor (URB597; 1 µM) and MAG lipase inhibitor (JZL184; 1 µM) that increase endogenous anandamide and 2-arachidonyl glycerol, respectively, excitatory current responses were potentiated. URB597-induced potentiation was blocked by CB1 antagonist AM-251 (2 µM). Collectively, this suggests somatostatin-positive oriens interneuron LTP is CB1-dependent.

## 1. Introduction 

Synaptic plasticity is a process whereby synapses can be strengthened or weakened by either presynaptically altering neurotransmitter release, or postsynaptically modifying synaptic receptor numbers. Plasticity is a critical attribute that allows for brain modification in an experience-dependent fashion, with the hippocampus encoding and consolidating memory. Two major synaptic plasticity forms exhibited in the brain are long-term potentiation (LTP) [1] and long-term depression (LTD) [2]. Hippocampal LTP of cornu ammonis (CA1) pyramidal cells is dependent on *N*-methyl-d-aspartate (NMDA) glutamate receptors, which induce LTP by triggering a signal cascade that results in the insertion of α-amino-3-hydroxy-5-methyl-4-isoxazolepropionic acid (AMPA) glutamate receptors into the postsynaptic membrane [3]. Other NMDA receptor-independent forms of plasticity also occur that are mediated by various mechanisms including lipid messengers known as endocannabinoids (eCBs).

Endocannabinoids are normally produced by catabolism of phosphatidylinositol 4,5-bisphophate from the lipid membrane. The various enzymes involved in the process include n-acylphosphatidylethanolamine phospholipase D (NAPE-PLD), diacylglycerol lipase alpha (DAGLα), and 12-lipoxygenase, which normally follow G-protein second messenger activation [4,5,6,7,8]. For example, activation of postsynaptic type I metabotropic glutamate receptors can result in formation of metabolites that are modified to produce eCBs or eicosanoid. These lipid molecules often act retrogradely on presynaptic receptors such as cannabinoid receptor 1 (CB1) to alter neurotransmission [9]. The two best-characterized and most widely expressed endocannabinoids are anandamide and 2-arachadonyl glycerol (2-AG). Anandamide is produced by several pathways, including by NAPE-PLD, and can bind to CB1, transient receptor potential vanilloid 1 (TRPV1) [6,10], and other G-protein-coupled receptors [11]. Anandamide is degraded by the enzyme fatty acid amide hydrolase (FAAH) [7,12]. 2-AG is produced in the brain by DAGLα and involved in retrograde signaling via CB1 [8]. Finally, the enzyme 12-lipoxygenase can synthesize 12-(*S*)-hydroperoxyeicosa-5*Z*,8*Z*,10*E*,14*Z*-tetraenoic acid (12-HPETE), which can activate TRPV1 receptors [13].

The hippocampus contains both excitatory and inhibitory neurons. Excitatory pyramidal cells are located in the stratum pyramidale layer and diverse inhibitory interneurons are present in layers such as the stratum radiatum and stratum oriens [14]. Hippocampal interneurons are a diverse group, and can be classified into several subtypes based on their function, axon projections, and firing pattern. These subtypes are often characterized by expression of calcium-binding proteins and neuropeptides, including somatostatin (SOM) and parvalbumin (PV). Hippocampal CA1 stratum oriens interneuron subtypes include oriens lacunosum-moleculare (O-LM) interneurons, which can be identified by the expression of somatostatin and have regular-to-fast action potential spiking patterns [15,16,17]. O-LM cell soma and dendrites reside in the stratum oriens and their axons project to the stratum lacunosum-moleculare layer. Another subtype is parvalbumin-positive basket cells, which project locally [18]. These basket cells are also involved in network oscillations in the hippocampus [19]. 

Stratum oriens interneurons exhibit plasticity, which is markedly unique compared to neighboring layers such as the stratum radiatum. For example, the type I mGluR (mGluR1 and mGluR5) agonist (*S*)-3,5-DHPG will depress glutamatergic synaptic responses onto radiatum interneurons [20], but potentiate glutamatergic synaptic responses onto oriens interneurons [21]. LTP is induced in O-LM neurons by high-frequency stimulus (HFS; 100 Hz) and is anti-Hebbian due to the requirement for postsynaptic hyperpolarization, which is needed to relieve a polyamine block of calcium-permeable AMPA receptors [22]. While this LTP is induced postsynaptically and depends on mGluRs, post-synaptic calcium, M1 muscarinic receptors, and alpha7 nicotinic acetylcholine receptors [23,24,25], it is expressed presynaptically. However, the full presynaptic mechanism has yet to be fully characterized and any potential involvement of eCBs in this plasticity is unknown, though it is independent of nitric oxide and TRPV1 presynaptic signaling [16]. Our goal was to determine whether interneuron LTP is dependent on presynaptic eCB signaling and whether interneurons have the machinery to produce these eCBs. While it is most common for eCB plasticity via CB1 receptors to induce LTD, CB1-dependent LTP was recently observed in both the hippocampal lateral perforant pathway [26] and in cortical inputs onto dentate granule cells [27]. Collectively, based on the role oriens interneurons play in feedback inhibition of CA1 pyramidal cells, and widespread network control including oscillations, and given that they undergo plasticity, it is important to fully characterize this novel form of LTP. Here, we report that LTP in stratum oriens interneurons is CB1-dependent and that oriens interneurons express eCB biosynthetic enzymes.

## 2. Results 

**Figure 1:**
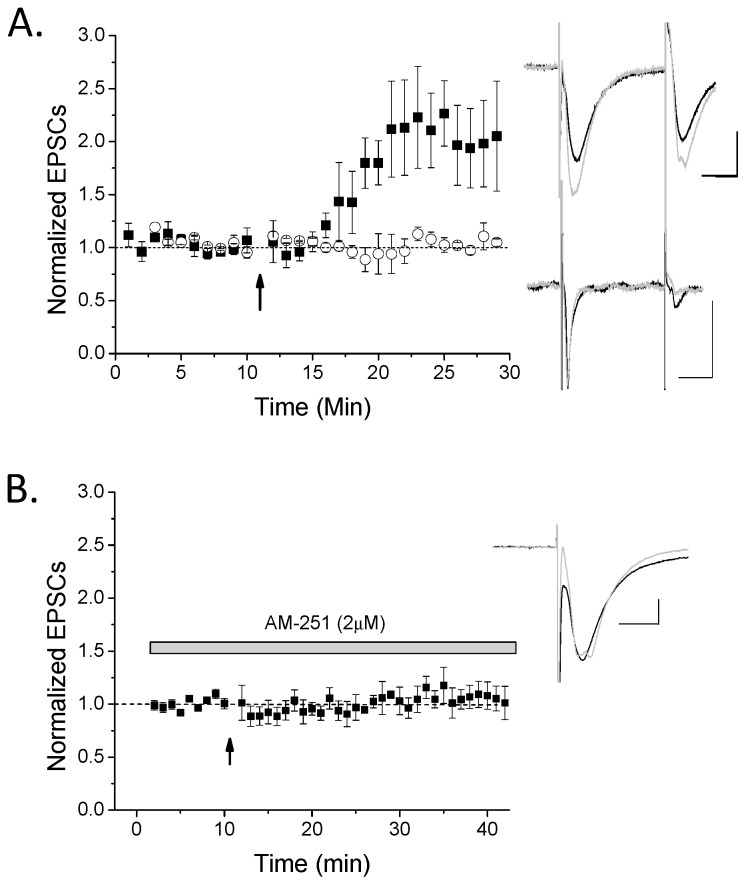
High-frequency stimulus long-term potentiation (HFS-LTP) only occurs in a subset of rat hippocampal oriens interneurons and is cannabinoid receptor (CB1)-dependent. (**A**) Oriens interneurons were recorded in whole cell voltage-clamp mode and 100 Hz high-frequency stimulus (HFS; arrow) while in current clamp mode was used to induce LTP. A majority (67%) of interneurons exhibited significant LTP (*n* = 8; ANOVA, *p* < 0.05), while a minority did not (*n* = 4; ANOVA, *p* > 0.05). ANOVAs were used to compare baseline to post-HFS excitatory postsynaptic currents (EPSCs) in order to group a cell having significant LTP versus no plasticity. These two groups (black symbols, LTP; white symbols, no plasticity) were significantly different from each other (*p* < 0.05, *t*-test). Inset: top traces are an LTP example and bottom traces are a no plasticity example. Traces were taken from 10–12 sweeps just before HFS (black) or ~10–15 min post-HFS (light gray). (**B**) This LTP was significantly blocked by AM-251 (*n* = 6; *p* < 0.05 compared to control LTP), suggesting it is CB1-dependent. Scale bars 100 pA, 10 msec. Plots, mean with s.e.m.

**Figure 2:**
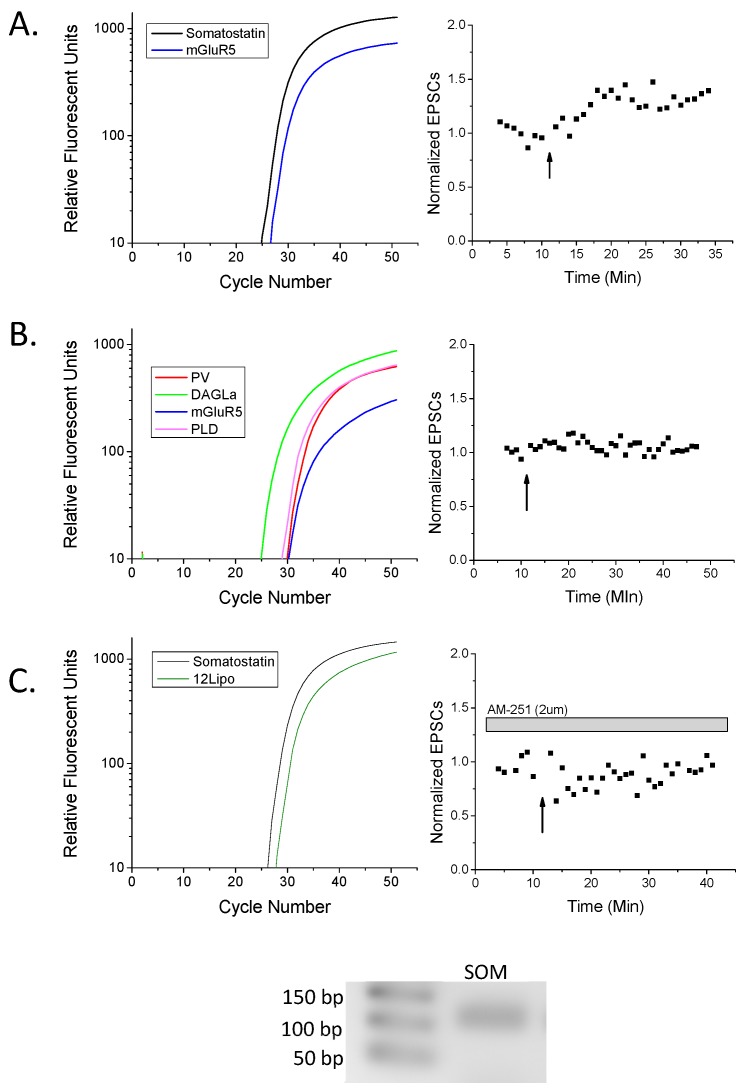
Real-time (RT)-PCR confirms somatostatin-positive (SOM+) rat hippocampal cells exhibit LTP. (**A**) An example SOM+ cell from Figure 1A that expressed type I metabotropic glutamate receptor 5 (mGluR5), and exhibited HFS-LTP. (**B**) An example parvalbumin-positive (PV+; somatostatin-negative) cell from Figure 1A that expressed diacylglycerol lipase (DAGLα), mGluR5, and n-acylphosphatidylethanolamine phospholipase D (NAPE-PLD, abbreviated PLD) that did not potentiate following HFS. (**C**) An example SOM+ neuron from Figure 1B expressing 12-lipoxygenase (12-lipo), that likely could have potentiated, but did not exhibit plasticity in the presence of the CB1 antagonist AM-251. Inset: Gel electrophoresis of the amplicon from the somatostatin (SOM) primers of the appropriate size with accompanying ladder in base pairs (bp). As a note, amplicons from all other primers have been published previously [10,29]. Trace color key: Black, somatostatin; blue mGluR5; lime green, DAGLα; red, parvalbumin; magenta, NAPE-PLD; forest green, 12-lipoxygenase.

The goal of our study was to investigate the role of endocannabinoid signaling in stratum oriens interneuron LTP. Therefore, we employed whole-cell patch clamp electrophysiology to identify plasticity of stratum oriens interneurons followed by real-time PCR (RT-PCR) on the extracted cell to examine mRNA components involved in endocannabinoid biosynthesis. To induce LTP, we applied a 100 Hz HFS to the stratum oriens layer near the recorded cell. LTP was evoked in 67% of interneurons (Figure 1A); the remaining cells evoked glutamate responses, but did not exhibit plasticity. To examine the involvement of eCBs and CB1 in this LTP, we applied the CB1 inverse agonist AM-251 to the slices for at least 15 min before LTP induction and observed LTP was blocked in all cases (Figure 1B). Controls with no HFS resulted in neither significant potentiation nor depression, and no change in EPSCs (data not shown; *n* = 6, *p* > 0.5). After the recording, cells were extracted in order to examine cellular mRNA. Classification of cells as interneurons was based on GAD65 and/or GAD67 mRNA expression and their location in the stratum oriens away from stratum pyramidale. In this way, interneurons were classified into four subtypes based on their expression of either somatostatin (SOM+), parvalbumin (PV+), calbindin (CB+), or calretinin (CR+) (see Table 1 and see examples in Figure 2). We further observed that in general, SOM+ cells were those exhibiting LTP in Figure 1 (Figure 2A), while SOM-negative cells, such as PV+ cells, did not (Figure 2B). Furthermore, LTP of SOM+ neurons was blocked in the presence of AM-251 (Figure 2C). Important to note here is that while the majority of LTP-exhibiting cells were SOM+ cells, one LTP cell was PV+, SOM-negative. However, we cannot determine whether this LTP cell was potentially a SOM-false negative cell, as some SOM+ cells also express PV [28], or a true PV+ basket cell. In addition, another LTP cell was included in the physiology though it was non-classifiable, likely due to false negatives for interneuron marker subtypes. One non-LTP cell was also unclassified. Collectively, the data demonstrate that CB1-dependent LTP mainly occurs in SOM+ cells. 

**Figure 3:**
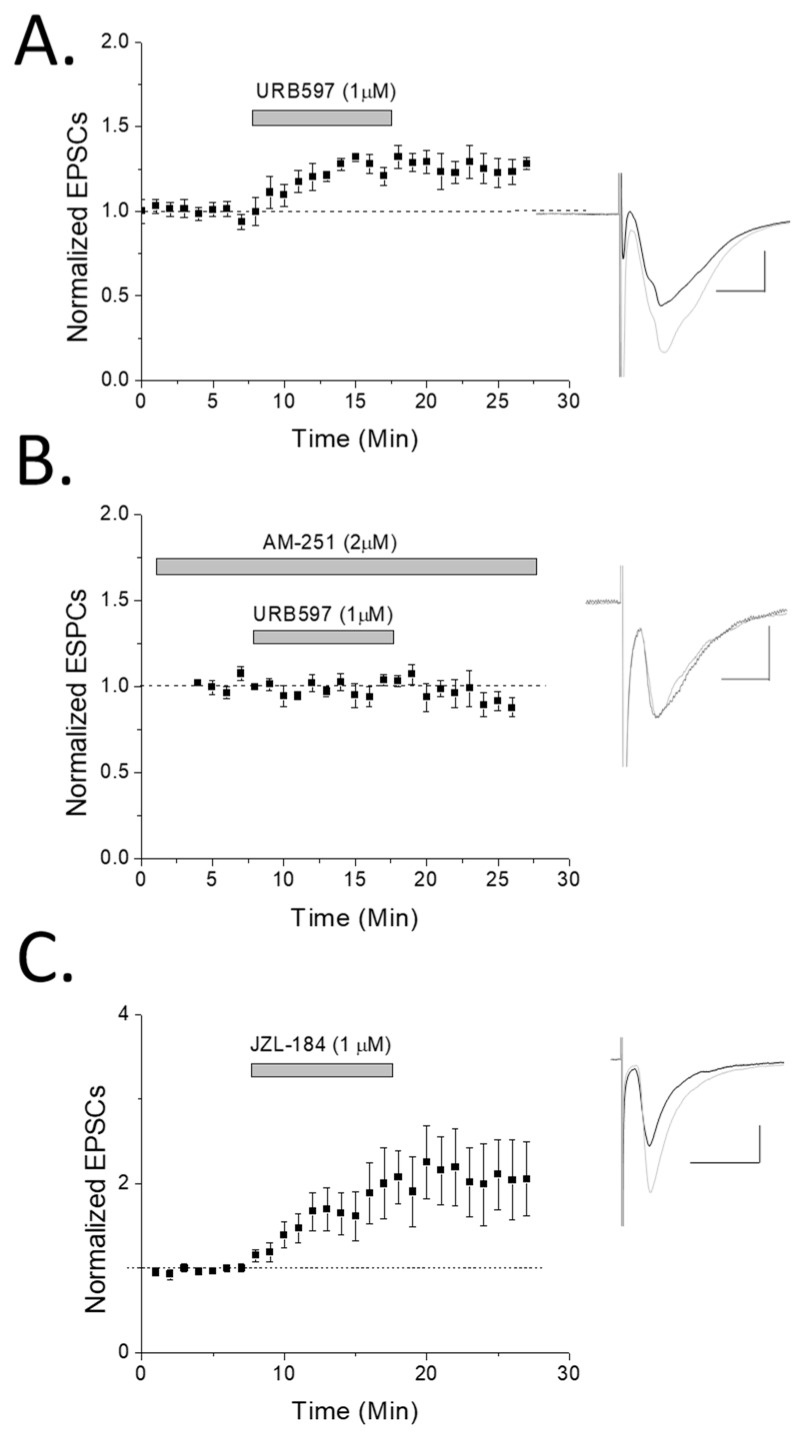
Fatty acid amide hydrolase (FAAH) inhibition potentiates rat hippocampal oriens interneurons in a CB1-dependent manner. (**A**) Stratum oriens interneurons were patched (*n* = 8) in whole cell voltage-clamp mode and recorded while the enzyme FAAH was inhibited by URB-597 (1 µM). A significant (*p* < 0.05; ANOVA) potentiating effect was observed, suggesting a role of anandamide in oriens synaptic plasticity. (**B**) CB1 antagonist AM-251 significantly blocked FAAH-induced potentiation (*n* = 7; *p* < 0.05; *t* test, comparing URB-597 to URB-597 + AM-251). (**C**) The monoacylglycerol lipase (MAG lipase) inhibitor, JZL184 (1 µM), used to prevent degradation of 2-AG also resulted in significant (*p* < 0.05; ANOVA; *n* = 5) enhancement of EPSC responses (individual cells were examined for significance in their potentiation by ANOVA and included if significant). Note that of the six significantly potentiating cells (of nine recorded from), four were confirmed SOM+ by posthoc PCR analysis while two were unclassifiable. Inset: traces were taken of 10–12 sweeps just before HFS (black) or ~15 min post-drug application (light gray).

Because CB1 receptors are normally involved in synaptic depression rather than potentiation, we wanted to confirm CB1 involvement in synaptic enhancements using a different approach. To do this, we recorded glutamate EPSCs from oriens interneurons before and after a fatty acid amide hydrolase (FAAH) inhibitor, URB597, was applied to the extracellular bath solution. FAAH inhibition prevents anandamide hydrolysis and increases synaptic levels of anandamide. We noted that FAAH inhibition produced a significant (*p* < 0.05) potentiation compared to baseline (Figure 3A). This potentiation was blocked by the CB1 antagonist AM-251 (Figure 3B), demonstrating CB1 dependence of anandamide-mediated synaptic enhancements. In addition, we applied the monoacylglycerol lipase (MAG lipase) inhibitor, JZL184 (1 µM), which prevents degradation of 2-AG. This resulted in significant (*p* < 0.05) enhancement of EPSC responses (Figure 3C) as well, supporting the FAAH inhibitor data and the involvement of endocannabinoids in potentiation of these excitatory inputs. 

To examine presynaptic location of LTP and FAAH-induced potentiation we performed paired pulse ratios as described previously [30] and noted both a trend for depressed ratios after HFS or URB597 application (data not shown; both had *p* < 0.1; see Figure 1A LTP traces). This suggested LTP is potentially presynaptic, which has been illustrated by many others examining oriens SOM+ interneuron LTP as described previously; thus, our data, while only trending, support that of others. 

As several groups, including ours, have data supporting the ability for various GABAergic interneurons, including those in the stratum radiatum, to produce eCBs and thus potentially modify their own activity, we examined this question using RT-PCR and immunohistochemistry (IHC). RT-PCR revealed that the most abundant interneuron, the SOM+ cells, expressed mRNA for DAGLα, NAPE-PLD, 12-lipoxygenase, and/or type I mGluRs (see Table 1). PV+ cells were the second-largest group, and expressed mRNA for NAPE-PLD, 12-lipoxygenase, and/or type I mGluRs, while CB+ cells had only one such cell expressing eCB enzyme mRNA. CR+ neurons were the minority, and did not express any of the eCB-related proteins we tested. This suggests stratum oriens interneurons, particularly SOM+ and PV+ cells, but not CB/CR cells, have the potential to produce eCBs. While we performed physiology on 33 cells where full PCR was carried out, not all were classifiable and were thus excluded from the RT-PCR expression data (*n* = 29 total, all of which expressed control 18S at a reliable level to confirm successful mRNA harvesting; Table 1). Lastly, while potential false negatives likely led to low expression levels of both eCB-producing enzymes and type I mGluRs (see discussion for further explanation), expression of DAGLα in some cells that exhibited LTP was noted. However, this alone cannot confirm interneuron DAGLα is used for CB1-dependent LTP, but alternatively could also be used for another purpose. 

**Figure 4:**
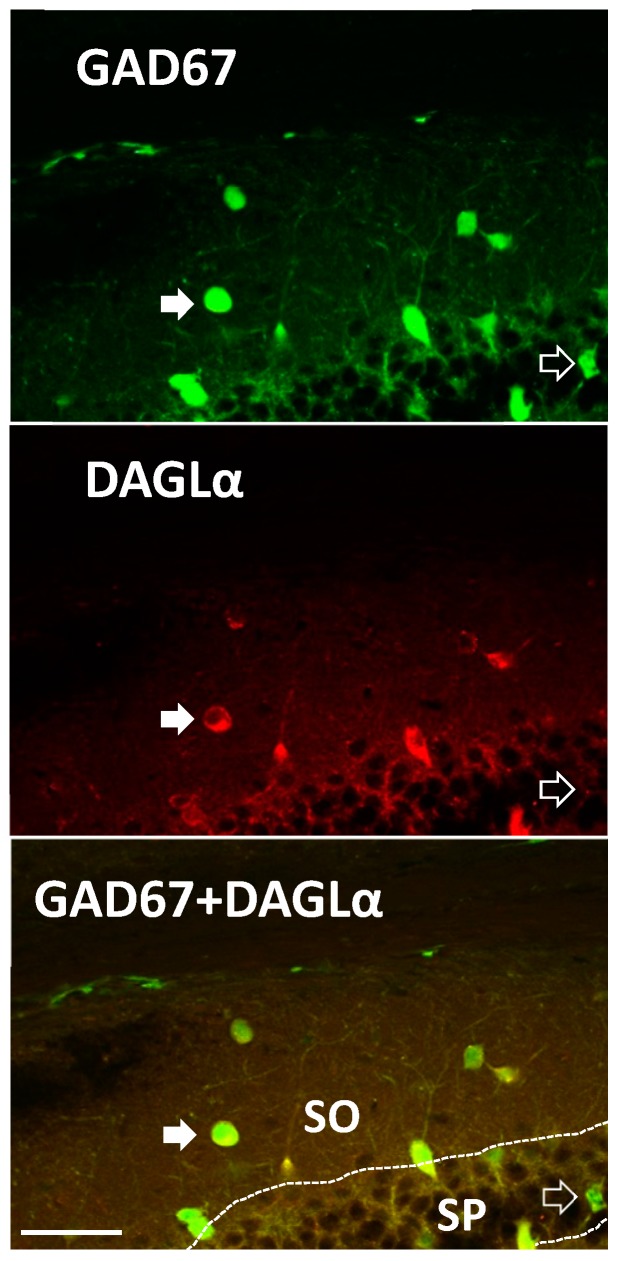
DAGLα is expressed in a subset of mouse hippocampal interneurons. GAD67-green fluorescent protein (GFP) knock-in mice were probed for DAGLα expression. We observed DAGLα immunoreactivity in the cytosol of GAD67-GFP stratum oriens (SO) interneurons and pyramidal cells of the stratum pyramidale (SP). Semi-quantitative analysis of co-labeling occurred in ~64% of GAD67-positive neurons in the same region as physiological recordings (*n* = 31 of 48; from slices in three different mouse brains of dorsal CA1). Closed arrows illustrate an interneuron that is double labeled with GAD67-GFP and DAGLα in the oriens. Open arrow denotes a GAD67-GFP labeled, DAGLα-negative neuron in the SP. GAD67-positive cells include populations of PV+ and SOM+ cells [31]. Scale Bar: 50 µm.

Lastly, to confirm that mRNA was actually translated to protein we employed IHC to examine protein expression of DAGLα, the enzyme producing the eCB 2-AG, within oriens interneurons of rodents where green fluorescent protein (GFP) was used as an interneuron marker. Mice, rather than rats, were used in this case as we employed a modified mouse line where GAD67-positive cells also express GFP, which was used in place of notoriously poor GAD antibodies, allowing us to make a positive identification of interneurons genetically. These GAD67-postive cells were demonstrated previously to include SOM+ and PV+ cells [31]. DAGLα exhibited cytosolic expression in 64% of GAD67-positive cells examined (Figure 4). Expression of DAGLα was noted in interneurons of the stratum radiatum as well. Collectively, our data indicate that SOM+ stratum oriens LTP is CB1-dependent, eCBs potentiate excitatory transmission to oriens interneurons, and SOM+ and PV+ interneurons have at least the potential to produce eCBs based on molecular studies.

## 3. Discussion 

This is the first demonstration of CB1-dependence for stratum oriens interneuron LTP and of eCB biosynthetic enzyme expression in oriens interneurons in a subtype-specific manner. Regarding interneurons, several subtypes in the oriens were identifiable based on their expression profile. The relative ratios of oriens interneuron subtypes shown here is also similar to what others have seen in the hippocampus [14]. For example, the majority cell type observed were SOM+, which are otherwise known as O-LM cells as their dendrites and soma are located in the oriens layer and their axon arborizes in the lacunosum-moleculare layer. SOM+ neurons have been previously shown to undergo LTP [32] in a manner that is dependent on type I mGluRs [32,33,34] which is consistent with our current findings. Using single-cell RT-PCR, we classified four main groups: SOM+, PV+, calretinin, and calbindin, as previously reported [14,31,35]. Collectively, we see that oriens interneurons can produce eCB synthetic proteins, and undergo CB1-dependent potentiation. 

### 3.1. eCB Production

Endocannabinoid signaling in the hippocampus has been previously studied [36], and has particular importance for stratum radiatum GABA cells [37]. Therefore, it is possible that PV+ and SOM+ oriens interneurons also participate in this type of signaling. Interneurons of stratum radiatum express mRNA for eCB biosynthetic enzymes [10], and produce eCBs that induce LTD to modify their own activity mediated by DAGLα [38]. Based on our data, oriens interneurons also appear to have the capacity to produce eCBs. The exception is CR-positive cells that are interneuron-selective innervating [39] as they are noted in this study and in the stratum radiatum [10] not to possess mRNA for eCB synthetic machinery. As a note, cells expressing CR and CB represented the minority of total cells (17%, 5 of 29 cells), which is consistent with previous reports [14,40]. Regarding PV+ and SOM+ neurons, these cells are differentially implicated in control of network hippocampal oscillations, theta-frequency, and gating information flow [41,42,43]. Our data revealed a similar proportion of these two cells as demonstrated by others [17,28]. 

Immunohistochemical staining revealed colocalization of DAGLα-positive neurons and GAD67-GFP in the stratum oriens, supporting our RT-PCR results. Using this GAD67-GFP mouse line, Tamamaki et al. observed GAD67-GFP in somatostatin-, parvalbumin-, and calretinin-positive neurons [31]. While we did not subcategorize GAD67 cells, because approximately two thirds of these cells expressed DAGLα and calretinin cells are less common, it is very likely that most of these were PV+ and/or SOM+ cells. While not all oriens interneurons express DAGLα, as noted by our RT-PCR and IHC, we do not know if this is of physiological consequence for eCB signaling in these interneurons or if this distinguishes subclasses of cells within these interneuron subtypes. These data also support previous findings of DAGLα in both excitatory and inhibitory neurons of the CA1, CA3, and dentate regions of the hippocampus [38], which further confirms immunohistochemistry results. It is also noteworthy that both PV+ and SOM+ cells express, for example, NAPE-PLD and DAGLα, however only SOM+ cells exhibit consistent LTP. Therefore, if eCB production was needed for LTP it is still possible that it is not produced in the oriens interneurons, but potentially from another local source such as pyramidal cells, which affect interneuron inputs. 

To produce eCBs, interneurons would also have to express type I mGluRs, which are normally required for eCB production [6,38,44]. Type I mGluRs are involved in various types of plasticity [20,21]. In addition, others demonstrate type I mGluR expression in O-LM and other oriens interneurons subtypes, and thus our data correlate well with these studies [21,28,45,46], though our results are at a lower percentage. This is likely because single cell RT-PCR techniques similar to ours often generate false negatives (up to ~30–40% depending on the target). Thus, the actual cellular expression of eCB enzymes and type I mGluRs are most likely higher than we note here. Therefore, while we are confident in the cells that did show positive target expression as we controlled for false positives, what we reported here are likely underestimates. Making interpretations of negative RT-PCR data is dubious as well, and so only positive data can give a reliable assessment. In conclusion, our data support others that oriens interneurons expressing type I mGluRs. 

### 3.2. CB1 eCB Plasticity

It was recently demonstrated that O-LM LTP was anti-Hebbian and employed a presynaptic mechanism [16] that requires activation of type I mGluRs [21,23], M1 muscarinic acetylcholine receptors [23], T-type calcium channels [24], and calcium-permeable AMPA receptors [47]. Nicholson et al. 2014 further demonstrated that the plasticity they observed was not dependent on TRPV1 nor nitric oxide, which are other mechanisms inducing presynaptic plasticity. Another common presynaptic receptor that we hypothesized could be involved in plasticity was CB1. However, hippocampal CA1 pyramidal cells whose recurrent collaterals innervate oriens interneurons reportedly do not express CB1 [48], and typically CB1 activation results in presynaptic LTD [49]. That being said, while CB1-dependent LTD is most common, CB1-dependent LTP was recently observed in the hippocampal lateral perforant pathway [26], as well as cortical inputs onto dentate granule cells [27]. These reports suggested that a CB1/integrin pathway increases neurotransmitter release and that CB1 works through a GTPase to modulate actin, which increases the amount of the readily releasable pool of vesicles. An alternative consideration to our data is that as CB1 is also expressed on septo-hippocampal inputs, cholinergic transmission in the hippocampus could be fine-tuned by endocannabinoid signaling, as noted previously [50]. As cholinergic signaling is required for oriens interneuron LTP [25], and M1 acetylcholine receptor inhibition [23] blocks this LTP, CB1 antagonism, in this case, could be altering acetylcholine levels and thus inhibiting LTP indirectly. While we do not know the mechanism of CB1-dependent LTP in the oriens or the location of CB1 receptor expression, it could be similar to one of these prior studies, or alternatively via CB1 at glutamatergic synapses coupling to a different intracellular signaling pathway. In summary, the CB1-containing inputs could be cortical in nature [27], from Schaffer Collaterals or alternatively present on another nearby synapse such as on cholinergic inputs that modulate glutamate input to oriens SOM+ interneurons. Additional studies are needed to confirm the location of CB1 receptors, and whether they are on glutamate and/or cholinergic terminals directly, and to genetically confirm CB1 involvement with CB1 knock-out mice in order to compare to the rats used in the present study. Indeed, these are matters of future and ongoing investigation. A final alternative is that increased anandamide levels could be causing a decrease in 2-AG levels, as observed in the striatum [51], thus, disinhibiting oriens interneurons. 

Finally, there are three last caveats to consider. The first one is that the LTP we observed had a slow onset, and a delay of about 3–4 min. This discrepancy compared to Le Duigou and Kullmann (2011) could be attributed to differences in methodology. For example, our pre- and post-conditioning recordings were performed in voltage-clamp, we utilized a cesium-based intracellular solution, and employed concentric rather than glass stimulating electrodes. Another caveat is that AM-251 at higher concentrations can block CB2 as well as mu opioid receptors in addition to CB1 at lower concentrations. However, several considerations support that AM-251 mediates its effect via CB1 receptors in this study. Firstly, AM-251 at 1–2 µM concentration has been used previously in brain slice hippocampal electrophysiology experiments and demonstrated to be specific for CB1 [52,53]. Secondly, while mu opioid receptors are expressed in the CA1 hippocampus, they do not bind endocannabinoids; therefore, the changes in potentiation by blockade of the enzyme FAAH that we note in our experiments, and the elimination of this potentiation by AM-251 (see Figure 3A,B), do not support mu opioid receptor involvement. In addition, mu opioid receptors are expressed presynaptically on GABA terminals of local interneurons [54] and thus blockade of GABA_A_ receptors eliminates mu opioid receptor effect on excitatory hippocampal activity [55]. As our experiments were all performed in the presence of GABA_A_ antagonist picrotoxin for both control LTP and with AM-251 where LTP is eliminated, AM-251 blockade of LTP is less likely via mu opioid receptors. The last caveat is that potentiation by FAAH and MAG lipase inhibitors does not necessarily directly correlate to LTP. LTP is a circuit phenomenon that requires the activation of multiple pathways, and this is simply eCB-induced synaptic glutamate potentiation. However, it does demonstrate that endocannabinoids can play a role in altering the activity levels of stratum oriens interneurons by themselves. 

## 4. Methods 

All experiments were performed in accordance with Institutional Animal Care and Use Committee protocols and followed the NIH guidelines for the care and use of laboratory animals. Male CD1 GAD67-GFP mice were used in immunohistochemical experiments. Male Sprague–Dawley rats age 15–34 days old were used for all electrophysiological experiments. 

### 4.1. Electrophysiology 

Rats were anesthetized with isoflurane and decapitated. Brains were removed and sectioned coronally on a vibratome at 400 µm. Recordings began at least one hour after cutting while tissue was stored in oxygenated artificial cerebral spinal fluid (ACSF) composed of 119 mM NaCl, 26 mM NaHCO_3_, 2.5 mM KCl, 1 mM NaH_2_PO_4_, 2.5 mM CaCl_2_, 1.3 mM MgSO_4_, and 11 mM glucose. 

Whole cell voltage-clamp experimental methods similar to these were described previously [10]. Briefly, hippocampal CA1 oriens cells were visualized using an Olympus BX51WI microscope (Center Valley, PA, USA) with a 40× water immersion objective. Cells were patched with a glass pipette filled with internal solution composed of 117 mM cesium gluconate, 2.8 mM NaCl, 20 mM HEPES, 5 mM MgCl_2_, and QX-314 (Tocris) (pH 7.28, 275–285 mOsm). Polyamines were omitted from the internal solution as done previously by others examining oriens interneuron LTP [16] so that calcium permeable AMPA receptors known to be required for LTP were not under voltage-dependent block. Picrotoxin (100 µM; Abcam) was added to the ACSF recording solution to block GABA_A_ receptor currents. AM-251, JZL184 and URB597 were purchased from Tocris (Bristol, UK). Cells were recorded in voltage-clamp at −65 mV. Stimulation to induce evoked synaptic transmitter release was accomplished using a concentric bipolar stimulating electrode placed in the stratum oriens layer near the cell recorded from to induce activation of glutamatergic axons including CA1 recurrent collaterals and potentially Schaffer Collateral inputs, along with other local neurotransmitter inputs. Stimulation ranged from 60 to 300 µA and evoked paired responses separated by 50 msec. Baseline and post-HFS stimulation was at 0.1 Hz for a 100 µsec duration to evoke EPSCs and the cell was recorded from for as long as possible. Baseline traces were acquired for approximately 10 min with no experiment going beyond ~12 min post-whole cell acquisition in order to avoid potential washout. Plasticity was induced using 2 stimulations at 100 Hz for 1 s (100 µsec stimulus duration), 20 s apart, while the cell was in current clamp mode, then recording resumed in voltage clamp mode. For all whole cell experiments, traces were recorded using Multiclamp 700B amplifier (Molecular Devices, Sunnyvale, CA, USA). Signals were filtered at 4 kHz and digitized with an Axon 1440A or 1550A digitizer (Molecular Devices) connected to a Dell personal computer with pClamp 10.2 or 10.5 Clampfit software (Molecular Devices). *p*-Values were obtained using a two-way unequal variance student’s *t*-test or ANOVA with *p* < 0.05 being considered significant, taken 10–15 min post-conditioning or drug application.

### 4.2. Reverse Transcription and Pre-Amplification 

RT-PCR methods were carried out similar to prior methods [10]. Briefly, cells used for RT-PCR analysis were extracted using gentle suction and placed into chilled reverse transcriptase reagents (BioRad, Hercules, CA, USA) and processed within 2 h. The entire cell was harvested to increase the amount of mRNA and thus reduce false negatives and attain as much starting mRNA as possible, as we had a large number of targets to examine. As a result, filling and post-hoc tracing of cells was untenable. One control sample of artificial cerebral spinal fluid was obtained for each slice and used to identify false positives due to contamination from extracellular mRNA, as detailed previously [29]. Using iScript cDNA Synthesis kit (BioRad), extracted cells were reverse transcribed to cDNA under the manufacturer’s protocol and cycled in a C1000 Thermocycler (BioRad) at 25 °C for 8 min, 42 °C for 60 min, and 70 °C for 15 min. Following reverse transcription, each cell was divided into three 5 µL aliquots, each of which received a different group of 10-fold diluted primers, iQ Supermix (BioRad), and ddH_2_O. The samples were then cycled in a C1000 Thermocycler (BioRad) starting at 95 °C for 3 min, then 15 cycles of 95 °C for 15 s, 57 °C for 20 s, and 72 °C for 25 s. Additionally, no template control multiplex tests were done to ensure there were no primer dimer or hairpin interactions among grouped multiplex primer sets that would interfere with later individual runs.

### 4.3. Primer Design 

Most primer and probe sequences were used in a prior study [10]. Primers were designed for somatostatin using the same parameters, efficiency, and melting temperature with Vector NTI software. The somatostatin forward primer, reverse primer, and probe sequences used were ACCCCAGACTCCGTCAGTTTC, GTTGGGCTCAGACAGCAGTTCT, and ACCGGGAAACAGGAACTGGCCAAGT, respectively. The appropriate fluorescent 6-carboxyfluorescein-tetramethylrhodamine (FAM-TAMRA) probe (Applied BioSystems, Inc., Waltham, MA, USA) for each target was included in the RT-PCR reaction to ensure specificity, as each individual probe was designed to bind only to its unique target amplicon, thereby eliminating alternative non-specific binding products of the primers from fluorescent analysis. Validation of these primers (gels, etc.) were illustrated in two prior publications [10,29]. Therefore, our RT-PCR data using specific probe-based fluorescence is more accurate in analysis than gels (which can still demonstrate non-specific products). As a positive reference/housekeeping control, we examined 18S to ensure successful harvesting of the cells. 18S values ranged from 14–18 on their cycle threshold (Ct) after multiplexing, similar to that reported by our lab previously [10,29]. If 18S was greater than 20, it was excluded from our analysis. Therefore, 18S PCR demonstrates successful completion of harvesting of mRNA, reverse transcription reaction, and multiplexing of these cells. In summary, 18S was used simply to confirm RT-PCR results of all other targets, but was not used to determine ΔCt values used to quantify differences in RT-PCR of these targets between cells, which we assume not to be significantly different from each other based on our prior study of stratum radiatum interneurons [10]. We illustrate raw RT-PCR Ct fluorescent curves from FAM-TAMRA probes to confirm expression of mRNA of these targets. Important to note is that we attempted to quantify expression of CB1 receptors as well, however as CB1 is the most highly expressed G-protein-coupled receptor in the brain it always came up in our ACSF controls as background, and therefore was eliminated from our analysis of single cells as a potential false positive. 

### 4.4. Quantitative RT-PCR Reaction 

Each pre-amplified cell was run for every target individually and in triplicate with its appropriate primer set and probe. Each cell was run in a CFX96 RT-PCR machine (BioRad) with a 95 °C hot start for 3 min, followed by 60 cycles of 95 °C for 15 s, 57 °C for 25 s, and 72 °C for 25 s. Cycle threshold values were determined with BioRad CFX manager 3.1 software. 

### 4.5. Immunohistochemistry

Male GAD67-GFP knock-in mice (20–30 days old) were used for immunohistochemical experiments. Animals were anesthetized with isoflurane and perfusion fixated (cardiac) with 0.9% NaCl, then 4% paraformaldehyde. The brains were removed and placed in paraformaldehyde where they remained overnight. They were then cryoprotected in 30% sucrose before being sectioned coronally through the hippocampus at 30 μm using a Microm HM 550 cryostat (Richard-Allan Scientific, Kalamazoo, MI). Free-floating sections were stored in 1 M PBS (pH 7.4), then washed in 0.2% Triton X in PBS for 30 min, a blocking solution of 1% BSA, 5% normal goat serum in PBS for 2 h, then in primary antibody overnight. The following day the sections were again washed in Triton X blocking solution, followed by the secondary antibody for 2 h. Slices were washed in 1 M tris buffered saline, and then mounted on non-frosted glass slides (Fisher Scientific, Pittsburgh, PA, USA) and cover-slipped using Fluoromount G (Southern Biotech, Birmingham, AL, USA). Images were taken using Olympus FluoView FV1000 confocal laser scanning microscope at 20× (Center Valley, PA, USA). The concentration of antibody used was DAGLα 1:500 (kindly provided by Dr. Ken Mackie), and AlexaFluor 350 goat anti-rabbit secondary 1:1000 (Invitrogen). An attempt to confirm NAPE-PLD expression by IHC was unsuccessful as we lacked confidence in the specificity of the antibodies we employed. 

## 5. Significance 

The results of the present study demonstrate hippocampal stratum oriens SOM+ cells exhibit CB1-dependent LTP. Further, a FAAH inhibitor potentiated glutamatergic inputs in a CB1-dependent manner. This is the first demonstration of CB1-dependent LTP in oriens interneurons, suggesting a broader and more variable role of CB1 and endocannabinoids in oriens plasticity. The diverse subtypes of oriens interneurons have various functions and projection patterns in the hippocampus. Understanding the plasticity of these individual subtypes is a further step in understanding hippocampal circuitry overall. For example, CB1 activation on feedforward interneurons in stratum radiatum inputs, when active, reduce GABA transmission to pyramidal cells and thus disinhibit pyramidal cells and increases pyramidal cell LTP [52], while CB1 activation in oriens induces potentiation of feedback interneurons to enhance GABA transmission, and thus potentially keep potentiated pyramidal cells from epileptic-type over-activation. Collectively, understanding the plasticity of oriens O-LM cells could play a functional role in the hippocampal output via CA1 pyramidal cells or modulating inputs from the perforant pathway in the lacunosum, where O-LM axonal arbors innervate. 

## Figures and Tables

**Table 1:** Summary of RT-PCR data. Individual stratum oriens interneurons from rat hippocampus were extracted for mRNA examination. Cells were sorted by subtype and the SOM+ cells were the most abundant, followed by parvalbumin, calbindin, and calretinin interneurons. Somatostatin and parvalbumin positive cells had the highest percent expression of mRNA for endocannabinoid/eicosanoid enzymes and type I mGluRs. The table represents the number of cells, from the total of each differentiated interneuron subtype as represented by the n value, with positive RT-PCR confirmation of endocannabinoid/eicosanoid-producing enzymes and type I mGluRs (i.e., 6 of 14 confirmed SOM+ cells tested positive of DAGLα mRNA).

eCB mRNA Expression in Oriens Interneurons				
Cell Marker	DAGLα	NAPE-PLD	12-Lipo	Type I mGluRs
SOM; *n* = 14	6	4	1	5
PV; *n* = 10	1	5	1	5
CB; *n* = 3	0	1	1	0
CR; *n* = 2	0	0	0	0
